# Feature Selection for Wearable Smartphone-Based Human Activity Recognition with Able bodied, Elderly, and Stroke Patients

**DOI:** 10.1371/journal.pone.0124414

**Published:** 2015-04-17

**Authors:** Nicole A. Capela, Edward D. Lemaire, Natalie Baddour

**Affiliations:** 1 Ottawa Hospital Research Institute, Ottawa, Canada; 2 Faculty of Medicine, University of Ottawa, Ottawa, Canada; 3 Department of Mechanical Engineering, University of Ottawa, Ottawa, Canada; University Hospitals of Geneva, SWITZERLAND

## Abstract

Human activity recognition (HAR), using wearable sensors, is a growing area with the potential to provide valuable information on patient mobility to rehabilitation specialists. Smartphones with accelerometer and gyroscope sensors are a convenient, minimally invasive, and low cost approach for mobility monitoring. HAR systems typically pre-process raw signals, segment the signals, and then extract features to be used in a classifier. Feature selection is a crucial step in the process to reduce potentially large data dimensionality and provide viable parameters to enable activity classification. Most HAR systems are customized to an individual research group, including a unique data set, classes, algorithms, and signal features. These data sets are obtained predominantly from able-bodied participants. In this paper, smartphone accelerometer and gyroscope sensor data were collected from populations that can benefit from human activity recognition: able-bodied, elderly, and stroke patients. Data from a consecutive sequence of 41 mobility tasks (18 different tasks) were collected for a total of 44 participants. Seventy-six signal features were calculated and subsets of these features were selected using three filter-based, classifier-independent, feature selection methods (Relief-F, Correlation-based Feature Selection, Fast Correlation Based Filter). The feature subsets were then evaluated using three generic classifiers (Naïve Bayes, Support Vector Machine, j48 Decision Tree). Common features were identified for all three populations, although the stroke population subset had some differences from both able-bodied and elderly sets. Evaluation with the three classifiers showed that the feature subsets produced similar or better accuracies than classification with the entire feature set. Therefore, since these feature subsets are classifier-independent, they should be useful for developing and improving HAR systems across and within populations.

## Introduction

Human activity monitoring and classification from wearable sensors can provide valuable information on patient mobility outside a hospital setting. While research in this area has received substantial attention in recent years, most research has involved able-bodied populations and proprietary hardware. An activity monitoring approach that works with ubiquitous technologies and is applicable across clinical populations would greatly benefit evidence-based decision making for people with mobility deficits.

Smartphones provide an ideal wearable computing environment that is convenient, easy to use, and rich with sensors, computing power, and storage. Many human activity recognition (HAR) systems have been developed for smartphone use [1], some using internal sensors and others interfacing with external biological sensors [2]. When measuring posture or movement, accelerometers and gyroscopes are popular choices since they are small, affordable, and easily worn on the body. Most commercial smartphones include accelerometers and gyroscopes, making them an ideal candidate for activity monitoring in real-world or rehabilitation settings.

Wearable sensors have been used to assess movement quality after stroke, such as upper extremity motion [3] or gait characteristics [4]. Activity levels, measured as the number of times total acceleration passes a threshold or minutes per day of activity, are typically collected using accelerometers or other body-worn sensors [5,6]. However, activity level analysis lacks contextual information. A system that provides contextual information on a person’s mobility activities would be of particular interest to healthcare professionals and researchers.

The typical signal processing steps for activity recognition are pre-processing, segmentation, feature extraction, dimensionality reduction (feature selection), and classification [7]. Features are raw data abstractions, usually calculated over a data segment or window (ex. signal magnitude area[8], correlations [8–10], interquartile range [11]). While numerous features can be extracted from a signal, increasing the number of features does not necessarily increase classifier accuracy since features may be redundant or not indicative of class (i.e., the activity being classified). Thus, feature selection is used to reduce data dimensionality and pass relevant and useful features to the classifier.

As Allen et al. remarked [12], many HAR approaches exist in the literature and each research group presents a particular data sample, defined classes, algorithm, and feature set. Therefore, extracting meaningful information to guide HAR algorithm development is difficult. Cheung et al. concluded that the most promising and practical activity classification solution would use a single, waist mounted, triaxial accelerometer, and future classifiers would be trained with larger samples from mobility impaired or older participants [13]. Smartphones with triaxial accelerometers meet the technical and practical hardware requirements but determining the best signal processing approach is still an open question. There is also uncertainty regarding the question of whether signal processing approaches need to be modified for different target populations since the majority of studies have involved able-bodied participants.

Cheung et al. [13] produced an extensive review of studies between 1980 and 2010 that used accelerometers to classify human movement. The majority of the 54 analyzed studies involved able-bodied participants. Nine studies involved patients who had various conditions, including Parkinson’s, back pain, and hemiparesis [13], and six studies involved elderly participants. One study involved cardiac rehabilitation patients [14]. These studies were limited by small sample sizes and used multiple sensors placed in various locations on the body, which can be obtrusive and inconvenient in a real life setting. Multiple sensors are unlikely to be consistently used in the community for long term monitoring.

A study with a larger data set (20 older adults and 32 Parkinson’s patients) evaluated a commercial activity monitor, the DynaPort MoveMonitor. This device achieved an accuracy of 65.1 to 98.9% for older adults and 57.5 to 96.9% for the Parkinson’s population, who had large variability [15]. Allen et al used a single triaxial accelerometer, mounted at the waist of six older healthy participants. A Gaussian mixture-model based system achieved mean accuracies of 77.3 to 98.9% [12]. Again, this study had a small sample size. Bidargaddi et al. used wavelet decomposition based measures to identify walking from other high intensity activities by using a triaxial accelerometer worn on the waist of cardiac outpatients. Sensitivity of 89.14% and specificity of 89.97% were achieved [14]. This study only differentiated walking from other activity states. A recent smartphone study with 20 younger people and 37 older people achieved total class sensitivity of 80.5% when trained on the older cohort and tested on the young, compared to a sensitivity of 69.2% when training on the young and testing on the older cohort [16]. This demonstrated the importance of considering differing populations while developing HAR systems.

From smartphone sensor data, many features have been identified for HAR [7]. Signal feature selection is necessary to identify the most important features and eliminate redundant features. A feature is considered statistically relevant if removing it decreases the prediction power, and a feature is considered redundant if another relevant feature exists with similar predicting power [17]. Feature selection methods can be categorized as filter methods, wrapper methods, or embedded methods [18].

Filter methods look at the data’s general characteristics to evaluate features without involving a classifier [17]. A wrapper method uses accuracy from a specific classifier to select features. Embedded methods incorporate feature selection as part of a classifier’s training process. Thus, both wrapper and embedded methods produce results that are specific to the classifier used for the task. Therefore, features weights, or feature subset selection, may only be useful to researchers using that particular classifier. In addition, the classifier depends on the training data set, which is relatively small for most HAR systems.

The purpose of this study was to determine signal features that are best suited for activity recognition using waist-worn smartphones with various populations, independent of the chosen classifier. This was achieved by examining a diverse dataset, using three different populations, and using various filter methods to select signal features independent of a classifier. Identifying feature subsets that improve activity classification will improve mobility monitoring models for use in future classifiers. Feature subsets with similar classifier performance to the full feature set should reduce computational burden, thus facilitating real-time implementations. This research is an important step in the larger aim of developing an accurate and robust HAR system for diverse populations.

## Materials and Methods

A convenience sample of 15 able-bodied participants, 17 participants over the age of 65, and 12 stroke patients were involved in this study (Table 1). The able bodied group were healthy students and staff at the Ottawa Hospital Rehabilitation Centre. The older participants were volunteers who were capable of completing the mobility tasks in the study. One senior participant walked with a limp and was awaiting surgery on their left leg. Another participant wore foot orthoses for the previous 2 years and had his patella replaced in 2013. He was also cautious on his left foot due to a bunion. Another participant had arthritis in their hip, which adversely affected walking gait. Seven stroke patients had left hemiparesis and three had right hemiparesis. Nine stroke patients had ischemic stroke and one had impairment because of a benign cerebral tumor. Two stroke patients used one crutch and one used an ankle-foot orthosis. All participants provided written informed consent and the study was approved by the Ottawa Health Science Network Research Ethics Board. Participant characteristics were recorded on a data sheet (i.e., age, sex, height, weight).

**Table 1:** Participant characteristics (mean and standard deviation).

Group	Number of Participants	Age (years)	Sex (% male)	Height (cm)	Weight (kg)
Able-body	15	26 ± 8.9	67	173.9 ± 11.4	68.9 ± 11.1
Elderly	17	74 ± 6.3	70	166.7 ± 9.3	69.6 ± 14.6
Stroke	12	54 ± 8.9	55	170.8 ± 5.5	82.7 ± 12.6

Participants performed a pre-determined set of daily living actions by moving through a continuous test circuit that included mobility activities (walking, standing, sitting, lying, ascending/descending stairs, ascending/descending ramps), daily living tasks (combing hair, brushing teeth, preparing food, eating, washing dishes), and environment changes (opening doors, using an elevator, traversing staircase landings, walking outdoors). Digital video was recorded while participants performed the activities, to establish a gold standard against which the sensor data could be compared. Activity timing was determined from the video and the video-based time was synchronized with smartphone sensor output by shaking the phone, thereby providing an easily recognizable accelerometer signal and video event.

Accelerometer and gyroscope data were collected using a Blackberry Z10 smartphone worn on the right-front hip and sampled at approximately 50Hz (smartphone sample rates vary, the Blackberry Z10 had a 3.84 Hz standard deviation [19]). Most modern smartphones contain comparable accelerometers and are capable of 50Hz or higher sampling rates [16,20]. The phone’s x and *y* axes were parallel to the phone`s face, with the *z*-axis pointed outward. Blackberry sensor data included raw acceleration (*x*, *y*, *z*), acceleration due to gravity (*x*, *y*, *z*), linear acceleration (raw acceleration minus gravity), and gyroscope data (*x*, *y*, *z*). The gravity signal (acceleration due to gravity) is calculated by the BlackBerry 10 operating system, using sensor fusion and a proprietary algorithm, and is used to determine the device’s true (gravity-free) linear acceleration. All data were collected using the TOHRC Data Logger application [21] and then imported into Matlab to calculate all features.

For HAR in this study, six activity classes were defined and labelled from the video recordings: sit, stand, lie, large movements (including walking, small steps, opening doors), stairs, and small movements (common activities of daily living that are often used in HAR studies [12,13,15]). The sensor data was continuous and thus contained sections between each identifiable class, which were labelled as transition states. For feature selection, the data were labelled by level of detail, such that each level contained only sensor data from a subset of classes. In this way, the selected features can be used in HAR systems that have varying detail levels. The levels were:

- Level 1: (2 classes)-Mobile, immobile (large movements and stairs labeled as mobile; sit, stand, lie, and small movements labeled as immobile)
- Level 2 (2 classes)-Sit, stand (not including small movements)
- Level 3 (3 classes)-Sit, stand, lie
- Level 4 (2 classes)-Large movements, stairs
- Level 5 (5 classes)-Ramp up, ramp down, large movements, stairs up, stairs down
- Level 6 (2 classes)-Small movements (Yes or No, only while sitting, standing or lying)
- Level7 (21 classes)-Transition states (transition between activities, listed in S1 Appendix)

Level 1 represented the lowest level of detail, and can be used for monitoring a person’s activity level without identifying individual activities. Level 2 differentiated between sit and stand, since these states are typically difficult to distinguish using a single waist worn accelerometer, causing them to be mutually misclassified [22,23]. Sit and stand activities with small movements, such as standing and working in the kitchen, were not included in level 2. Level 3 represented the three common immobile states (sit, stand, lie), including small movements in these states. Level 4 separated mobile states into large movements (predominantly walking) and stairs. Ramps were not included in level 4 since the sensor signals are typically similar to level walking. Level 5 represented the highest level of detail, including level walking, ramps, and stairs, to investigate features that can be considered when differentiating between activities that can have similar signals. Level 6 represented small movements, since these are difficult to detect using a waist worn sensor. Level 7 represented transitions between states.

### Features

Time domain features are typically used in HAR systems because they help preserve battery life by virtue of being less computationally intensive [24]. Seventy six features (Table 2) were selected from the literature and from observation of accelerometer and gyroscope pilot test data. These features were calculated over short sliding windows (1 second, no overlap) to allow a fast response in real time and to improve detection of short duration movements [12].

**Table 2:** Features derived from raw sensor data.

Feature #	Feature Description
1	Sum of range of linear acceleration
2	Sum of standard deviation of linear acceleration
3	Simple moving average of sum of range of linear acceleration
4	Difference to *y* (*y*_gravity_—*z*_gravity_—*x*_gravity_)
5, 6, 7	Range of gravity vector (*x*, *y*, *z*)
8,9,10	Mean of gravity vector (*x*, *y*, *z*)
11,12,13	Range of linear acceleration (*x*, *y*, *z*)
14,15,16	Mean of linear acceleration (*x*, *y*, *z*)
17,18,19	Kurtosis of gravity vector (*x*, *y*, *z*)
20	Sum of Kurtosis of gravity vector
21	Sum of standard deviation of linear acceleration
22	Sum of variances gravity (summed diagonal of covariance matrix)
23	Simple moving average of sum of variances
24	Maximum slope of simple moving average of sum of variances
25–30	Covariance matrix elements of gravity vector
31–36	Covariance matrix elements of linear acceleration vector
37–42	Covariance matrix elements of rotated linear acceleration vector [25]
43	Velocity from integral of rotated linear acceleration^1^ (excluding *y*-axis)
44	Velocity for *y*-axis
45,46,47	Mean Euclidean norm (Linear, rotated linear^1^, raw acceleration)
48,49,50	Skewness of rotated linear acceleration (*x*, *y*, *z*)^1^
51,52,53	Moving average of skewness of rotated linear acceleration (*x*, *y*, *z*)^1^
54	Sum of moving average of skewness (*x*, *y*)
55	Range of rotated linear acceleration (*x*)
56	Moving average of distance from rotated linear acceleration^1^
57,58,59	Mean absolute linear acceleration (*x*, *y*, *z*)
60,61,62	Harmonic mean linear acceleration (*x*, *y*, *z*)
63,64,65	Cumulative sum linear acceleration (*x*, *y*, *z*)
66	Correlation between acceleration along gravity and heading
67	Average velocity in gravity direction
68	Average velocity in heading direction
69,70,71	Gyroscope mean (*x*, *y*, *z*)
72,73,74	Interquartile range linear acceleration (*x*, *y*, *z*)
75	Zero cross rate
76	Mean cross rate

Feature files were generated from the sensor data and class files were created using the activities identified in the video recordings, and synchronized with the feature files. Each 1 second window was considered an occurrence. For example, sitting for 5 seconds was considered 5 occurrences. When segmenting the data, a 2 second window was selected on either side of a change of state to encompass the transition features; therefore, transition features were not included in the feature selection process for the surrounding states. Class distributions at each level are shown in Table 3. Since this is a realistic data sample representing activities of daily living, class imbalances occur. For example, there were more instances of walking or sitting than climbing stairs or lying down.

**Table 3:** Class distributions at each level.

Level	Class	Number of instances
1	Immobile	12841
1	Mobile	17607
2	Stand	4072
2	Sit	2756
3	Stand	8649
3	Sit	2808
3	Lie	1353
4	Large Movements	16720
4	Stairs	887
5	Large Movements	15262
5	Stairs up	582
5	Stairs down	458
5	Ramp up	438
5	Ramp down	346
6	Small moves	4577
6	None	8233

With a phone positioned on the front of the pelvis, the phone’s orientation when the person is standing upright differs depending on the individual’s body type or clothing. To address this, a quaternion based rotation matrix method was used to correct for these differences [25]. A ten second sample of accelerometer data was collected while the participant was standing still. One second of this sample with the smallest standard deviation was used to calculate the rotation matrix constants. The remaining raw linear accelerometer data were multiplied by this matrix to create a consistent linear acceleration signal that was corrected for initial phone orientation.

### Feature Selection

The current study focused on filter methods for feature selection, since these methods are independent of the selected classifier. Three filter methods were chosen: Relief-F, Correlation-based Feature Selection (CFS), and Fast Correlation Based Filter (FCBF). These feature selection methods do not rely on information theory, which can be biased towards features based on activities that occur more often in the data set.

Relief F is a commonly used filter method that ranks features by weighting them based on quality (relevance). For each instance, the algorithm finds the nearest hit (data point from same class) and nearest misses (data points from different classes). Feature relevance is based on how well instances from different classes and instances from the same class are distinguished [17,26]. Rather than providing a subset of features, Relief-F weights all features according to relevance. The formula used to update the weight of each feature is Eq (1).

$$w_{i}={\sum_{j=1}^{N}\left(x_{i}{}^{j}-nearmiss{\left(x^{j}\right)}_{i}\right)}^{2}-{\left(x_{i}{}^{j}-nearhit{\left(x^{j}\right)}_{i}\right)}^{2}$$ (1)

where *w* is the weight of the *i* ^th^ feature, $x_{i}^{j}$ is the value of the *i* ^th^ feature for point *x* ^*j*^, and *N* is the total number of data points. Nearhit *x* ^*j*^ and nearmiss *x* ^*j*^ are the nearest data point to *x* ^*j*^ in the same and different classes, respectively [17].

One concern with Relief-F is that it does not evaluate redundancy in comparison to other features [17]. However, Relief-F has been reported to be useful in cases with strong interdependencies between fields [26]. Since Relief-F does not select a subset of features, an appropriate number of features to include in each subset was determined by processing the ranked feature list with three common classifiers (Naïve Bayes, Support Vector Machine (SVM), j48 Decision tree (j48)) using every possible number of features, added in order of rank. For all but 3 cases, the accuracy achieved using the ten highest ranked features was within 5% of the maximum accuracy achieved. Thus, subsets of the top 10 ranked features were used to compare populations.

Correlation based Feature Selection (CFS) evaluates the relevance of features from a correlation based heuristic that examines inter-correlation among features along with their ability to predict classes [27]. Thus, CFS selects features that are highly correlated with the class and uncorrelated with each other. Feature relevance is quantified using Eq (2).

$$merit_{s}=\frac{k\bar{r_{cf}}}{\sqrt{k+k(k-1)\bar{r_{ff}}}}$$ (2)

The subset *S* contains *k* features, $\bar{r_{ff}}$ is the average feature correlation, and $\bar{r_{cf}}$ is the mean feature-class correlation. This equation is a version of Pearson’s correlation with standardized variables. CFS uses a “forward best first search with a stopping criterion of five consecutive fully expanded non-improving subsets” [27].

The Fast Correlation Based Filter (FCBF) method evaluates feature merit by examining the predominant correlation between features and classes and selecting the predominant features from redundant peers. By using subsets of features based on symmetrical uncertainty, the algorithm can more efficiently analyze feature redundancy to perform a faster selection and achieve a high level of dimensionality reduction (selecting a small number of features) [28].

These three filter methods were chosen because they deal with potential issues when selecting multiple features from a common data set. Specifically, Relief F is useful in cases with strong interdependencies between fields and, since the features were derived primarily from the same accelerometer sensor data, interdependencies could occur. CFS selects features that are highly correlated with the class and uncorrelated with each other, which is desirable. Since the features were expected to correlate with each other, it is necessary to identify features that can be used together to increase performance, without being redundant. FCBF also compares correlations between features, yet tends to select smaller subsets than CFS. This is ideal because reducing computational cost by using fewer features is beneficial in a wearable system.

CFS, FCBF, and Relief-F filter methods were run in Matlab using the Arizona State University Feature Selection repository [17]. The algorithms were executed for each level and each population.

### Evaluation of Feature Selection

To evaluate whether the feature subsets were more effective for classification than the entire feature set, three common classifiers were run using all features and then using the feature subsets: Naïve Bayes, SVM, and j48 from the Arizona State University Feature Selection repository [17]. For each population, leave-one out cross validation was performed (as in [16]) at each level. Each level contained a subset of data from all participants. Data from all but one participant were used to train the classifier, which was then tested on data from the one “left out” participant. This was repeated for each participant (i.e., cross validation) to generate an accuracy data set for statistical analysis. A paired samples sign test was used to identify significant differences in classifier accuracy between the full feature set and feature subsets (p<0.05), since the data were neither symmetrical nor normal. The Benjamini–Hochberg procedure was used to correct for multiple comparisons. This classification procedure evaluated the feature selection results and provided outcome measures to determine if the subsets should be implemented in a HAR system.

## Results

The selected features for each population are shown in Table 4, 5, and 6. In general, the CFS method selected larger subsets that contained between 2 and 22 features, while FCBF selected subsets with 1 to 11 features.

**Table 4:** Features selected for able bodied participants.

Level	CFS	FCBF	Relief-F
1	3, 6, 12, 23, 27, 39, 43, 64, 66, 70, 71, 73	3, 6, 27, 39, 64, 66	1, 2, 10, 11, 12, 15, 21, 33, 54, 58
2	10, 18, 23, 47	10, 18, 23, 47	7, 12, 18, 20,23, 52, 58, 65, 66, 73,
3	4, 9, 10, 42, 68	4, 9, 10, 42, 68	17, 34, 42, 44, 52, 57, 60, 61, 712, 76
4	3, 4, 7, 8, 14, 15, 23, 24, 25, 29, 34, 42, 48, 52, 57, 68	1, 8, 14, 15, 17, 29, 52	35, 48, 51, 52, 57, 60, 65, 66, 70, 75
5	4, 5, 7, 10, 23, 28, 29, 39, 42, 56	4, 20, 28, 39	11, 16, 29,36, 42, 59, 64, 76
6	3, 4, 9, 10, 11, 35, 37, 47, 48, 52, 56, 61, 712, 64, 70	10, 37	13, 44, 49, 46, 48, 52, 58, 65, 72, 76
7	3, 23, 24, 47, 68	8	7, 11, 17, 18, 20, 48, 49, 50, 55, 75

**Table 5:** Features selected for senior participants.

Level	CFS	FCBF	Relief-F
1	3, 7, 12, 22, 23, 25, 33, 35, 38, 44, 44, 57, 55, 56, 58, 58, 64, 66, 70, 71, 73, 74	25, 56, 58	15, 16,19, 44, 56,57, 59, 65, 66, 75
2	4, 10, 15, 24, 44	10, 15, 24, 28	1, 2, 3, 7, 11, 27, 30, 66, 67, 71
3	4, 10, 42, 45	4, 10, 42	16, 32, 47, 57, 56, 58,712, 64, 65, 76
4	4, 9, 10, 15, 20, 23, 24, 25, 27, 29, 38, 41, 44, 47, 48, 49, 52, 54, 66, 68	4, 29, 39, 44, 52, 61 63, 66	1, 12, 14, 18, 49, 50, 52, 63, 66, 76
5	4, 22, 24, 25, 29, 43, 44, 49, 52, 54, 66	4, 29, 42, 43, 44, 52	12, 14, 25, 40, 42, 63, 64, 67, 71, 72
6	3, 4, 8, 10, 11, 51, 56, 60, 61, 70, 72, 76	4, 11	22, 27, 43, 57, 56, 66, 68, 69, 73, 75
7	9, 44, 45, 48, 51, 72	4	13, 14, 15, 19, 40, 44, 50, 64, 67, 72

**Table 6:** Features selected for stroke participants.

Level	CFS	FCBF	Relief-F
1	3, 5, 6, 23, 25, 32, 38, 43, 49, 57, 56, 61, 68, 69, 70, 71, 74, 76	3, 25, 27, 32, 57, 61, 63, 65, 66, 76	7, 45, 48, 52, 54, 55, 56, 61, 66, 68
2	9, 10, 38, 47, 66, 68	26, 66, 70	1, 2, 5, 11, 12, 13, 14, 15, 28
3	4, 10, 44, 67 726:	10, 44	17, 18, 20, 44, 50, 51, 57, 64, 65
4	4, 18, 24, 29, 31, 47, 48, 52, 56	18, 24, 52, 56	14, 17, 19, 35, 37, 39, 41, 48, 63
5	3, 4, 7, 10, 16, 17, 18, 20, 24, 25, 29, 39, 47, 48, 47, 54, 56, 58, 59	10, 20, 29, 39, 63	2, 5, 21, 33, 34, 49, 46, 59, 74
6	10, 30, 47, 48, 67, 70, 72	70, 76	15, 17, 18, 20, 44, 49, 51, 57, 65
7	47, 48	30	11, 15, 43, 44, 49, 55, 58, 72, 76

### Features Selected by Population

For the able-bodied group (Table 4), features selected by CFS and FCBF methods were similar. Often, the FCBF features were a subset of the features selected by CFS (levels 1, 6). This was expected since these algorithms are similar. Features 18 and 23 (*y* gravity kurtosis and simple moving average of sum of gravity variances, respectively) were selected by all three algorithms to separate sitting and standing states. Only feature 42 (element (2, 3) of the rotated linear acceleration covariance matrix) was selected by all three algorithms to distinguish sitting, standing, and lying states, and feature 52 (moving average skewness of rotated Y linear acceleration) was selected by all three algorithms to determine if the person was climbing stairs or walking. No feature was selected by all algorithms for differentiating between mobile and immobile states, although feature 12 (range of *y* linear acceleration) was selected by both Relief-F and CFS.

For elderly participants (Table 5), the features selected by FCBF were subsets of the features selected by CFS for levels 1, 3, and 6, with only one extra feature selected when separating sitting and standing (feature 28: element (1,2) of the gravity covariance matrix) and when identifying the mobile state at level 5 (feature 42). All three algorithms selected feature 56 (moving average of distance from rotated linear acceleration) to differentiate mobile and immobile states, and feature 52 to discriminate other large movements from stairs.

For stroke participants (Table 6), all algorithms selected feature 61 (harmonic mean *y* linear acceleration) to distinguish mobile and immobile states, and feature 44 (*y* velocity) to differentiate sitting, standing, and lying down. The stroke group had the least agreement between algorithms for selected features.

Table 7 compares common features selected across populations by CFS. The CFS algorithm selected common features for all populations at every detail level, except for transitions. Table 8 shows the classifier accuracy for all features and the feature subset from CFS. Classifier performance was unchanged or significantly improved when using the selected feature subsets, meaning that redundant features were eliminated without sacrificing classification accuracy. As an example, Table 9 shows confusion tables for each classifier, run on the entire Level 3 dataset (all populations) using all features and using the CFS feature subset. For level 3, it can be considered that the cost of misclassifying a sitting state as standing is higher than the cost of misclassifying a sitting state as lying down, since lying down and sitting are both sedentary states with little energy expenditure.

**Table 7:** Common features selected across populations using CFS.

Level 1	Level 2	Level 3	Level 4	Level 5	Level 6	Level 7
**Able bodied and Senior**						
3, 12, 23, 64, 66, 70, 71, 73	10	4, 10, 42	4, 15, 23, 24, 25, 29, 52, 68	4, 29	3, 4, 10, 11, 56, 61, 70	n/a
**Able bodied and stroke patients**						
3, 6, 23, 43, 70, 71	10	4, 10	4, 24, 29, 52	4, 7, 10, 29, 39, 56	10, 70	n/a
**Stroke patients and senior**						
3, 23, 25, 38, 57, 56, 70, 71, 74	10	4, 10	4, 24, 29, 52	4, 24, 25, 29, 54	10, 70, 72	n/a
**All three populations**						
3, 23, 70, 71	10	4, 10	4, 24, 29, 52	4, 29	10, 70	n/a

**Table 8:** Classifier accuracy with all features and with selected feature subset using CFS.

	Features	Level 1	Level 2	Level 3	Level 4	Level 5	Level 6	Level 7
Able bodied								
Bayes	All	97.31	94.93	77.93	72.32	85.58	78.16	20.11
	Selected	97.52	95.62	94.42	81.94	94.32	82.72	21.87
	Sig	0.146	0.754	**0.001**	**0.001**	**0.013**	0.118	0.180
SVM	All	63.62	74.05	73.47	84.97	94.90	72.06	22.50
	Selected	86.79	96.70	88.12	84.97	94.90	78.72	20.25
	Sig	**< 0.001**	**< 0.001**	**0.001**	1.000	1.000	0.180	0.549
j48	All	94.69	96.85	95.02	71.38	90.52	78.16	20.36
	Selected	97.27	96.40	95.07	75.24	92.80	81.97	22.04
	Sig	0.581	1.000	0.109	0.035	1.000	0.302	0.791
Senior								
Bayes	All	94.45	86.75	79.02	76.57	91.57	82.61	17.85
	Selected	95.08	85.54	88.88	89.56	94.73	87.44	24.50
	Sig	**0.001**	1.000	**0.002**	**< 0.001**	**< 0.001**	**< 0.001**	**< 0.001**
SVM	All	65.57	67.81	70.14	91.07	94.49	67.14	18.38
	Selected	88.42	77.82	70.45	91.07	94.51	63.35	21.48
	Sig	**< 0.001**	0.143	0.210	1.000	1.000	0.143	0.332
j48	All	94.37	81.12	87.31	85.39	93.92	84.05	22.84
	Selected	94.78	80.87	85.87	87.68	94.55	83.51	23.19
	Sig	0.143	0.454	0.049	0.210	0.629	0.629	0.804
Stroke								
Bayes	All	96.18	76.35	77.88	74.31	83.05	75.42	26.98
	Selected	96.49	82.49	86.61	85.66	90.69	83.18	29.52
	Sig	0.039	0.180	0.012	0.012	0.012	0.227	0.344
SVM	All	67.14	61.02	66.38	90.59	95.97	64.09	26.58
	Selected	92.07	55.57	65.35	90.59	95.97	66.85	29.73
	Sig	**0.001**	0.125	1.000	1.000	1.000	1.000	0.065
j48	All	94.56	81.63	82.90	81.87	93.00	79.52	22.09
	Selected	95.20	84.07	80.23	85.89	94.76	77.37	23.40
	Sig	0.549	1.000	1.000	0.549	1.000	1.000	0.549
All Populations								
Bayes	All	95.62	84.81	78.36	76.74	87.18	78.26	11.97
	Selected	96.02	85.99	89.00	84.19	92.17	84.01	22.28
	Sig	**< 0.001**	0.617	0.165	1.000	1.000	**< 0.001**	0.522
SVM	All	66.90	68.38	70.76	88.82	95.01	68.59	21.35
	Selected	90.59	67.59	72.82	88.82	95.01	77.99	21.51
	Sig	0.029	1.000	0.118	0.874	0.871	0.877	0.127
j48	All	94.97	86.20	89.53	83.02	92.65	81.83	20.98
	Selected	95.84	85.30	88.52	84.01	93.16	82.44	20.12
	Sig	0.549	1.000	0.754	0.065	0.549	1.000	0.227

Bold cells show significant differences after correction for multiple tests.

**Table 9:** Confusion Tables for all populations at Level 3 using all features and feature subsets selected by CFS (each instance represents 1 second).

		Bayes			SVM			j48		
Features	Class	Stand	Sit	Lie	Stand	Sit	Lie	Stand	Sit	Lie
All	Stand	2135	219	7	2576	468	221	2522	52	1
	Sit	441	635	0	0	388	0	54	804	2
	Lie	0	2	404	0	0	190	0	0	408
CFS subset	Stand	2526	240	0	2546	47	8	2544	72	1
	Sit	50	612	3	30	809	0	32	784	1
	Lie	0	4	408	0	0	403	0	0	409

Table 10 shows the common features selected between populations by the FCBF algorithm and Table 11 shows the classifier accuracy for all features and the FCBF feature subset. Since FCBF subsets were smaller, there were fewer common features between populations than CFS. Four of 168 cases showed decreased classifier performance, though three of the accuracy differences were 1.6% or less, which is not clinically significant. The other case was a change from 22.50 to 17.41% accuracy; however, both results were low and likely unacceptable for making decisions on a person’s mobility status. These results were from the transition data set (level 7), which was not well identified using any of the classifiers. All other cases showed unchanged or significantly improved classifier performance, demonstrating that redundant features were eliminated without sacrificing classification accuracy.

**Table 10:** Common features between populations using FCBF.

Level 1	Level 2	Level 3	Level 4	Level 5	Level 6	Level 7
**Able bodied and Senior**						
n/a	10	10, 4, 42	29, 52	4	n/a	n/a
**Able bodied and stroke patients**						
3, 27, 66	n/a	10	52	39, 20	n/a	n/a
**Stroke patients and senior**						
25	n/a	10	52	29	n/a	n/a
**All three populations**						
n/a	n/a	10	52	n/a	n/a	n/a

**Table 11:** Classifier accuracy with all features and with selected feature subset using FCBF.

	Features	Level 1	Level 2	Level 3	Level 4	Level 5	Level 6	Level 7
Able bodied								
Bayes	All	97.31	94.93	77.93	72.32	85.58	78.16	20.11
	Selected	97.43	95.62	94.42	82.90	94.83	78.01	21.26
	Sig	0.549	0.754	**0.001**	**0.001**	0.035	0.607	0.791
SVM	All	63.62	74.05	73.47	84.97	94.90	72.06	22.50
	Selected	82.84	96.70	88.12	84.56	94.70	72.05	17.41
	Sig	**< 0.001**	**< 0.001**	**0.001**	0.031	0.031	1.000	**< 0.001**
j48	All	94.69	96.85	95.02	71.38	90.52	78.16	20.36
	Selected	97.12	96.40	95.07	77.80	93.73	77.84	16.24
	Sig	0.581	1.000	0.109	0.180	0.302	0.791	0.607
Senior								
Bayes	All	94.45	86.75	79.02	76.57	91.57	82.61	17.85
	Selected	95.51	84.24	88.88	92.09	95.82	85.75	24.49
	Sig	**0.013**	1.000	**0.002**	**< 0.001**	**< 0.001**	0.049	0.210
SVM	All	65.57	67.81	70.14	91.07	94.49	67.14	18.38
	Selected	88.51	72.37	70.45	91.07	94.30	62.79	21.13
	Sig	**< 0.001**	0.454	0.210	1.000	0.039	0.332	0.332
j48	All	94.37	81.12	87.31	85.39	93.92	84.05	22.84
	Selected	94.93	86.52	85.87	87.56	93.96	84.23	19.57
	Sig	0.077	0.332	0.049	0.332	1.000	1.000	0.210
Stroke								
Bayes	All	96.18	76.35	77.88	74.31	83.05	75.42	26.98
	Selected	96.72	76.34	81.34	88.17	95.35	75.80	31.48
	Sig	0.021	0.227	1.000	**0.001**	**0.001**	1.000	0.109
SVM	All	67.14	61.02	66.38	90.59	95.97	64.09	26.58
	Selected	77.31	70.81	64.75	88.97	95.95	73.40	24.57
	Sig	**0.001**	0.344	0.549	**0.001**	0.109	0.065	0.549
j48	All	94.56	81.63	82.90	81.87	93.00	79.52	22.09
	Selected	95.66	88.13	79.61	87.66	94.32	76.96	24.76
	Sig	1.000	0.754	0.065	0.549	1.000	1.000	0.227
All populations								
Bayes	All	95.62	84.81	78.36	76.74	87.18	78.26	11.97
	Selected	96.27	80.75	88.09	88.88	94.29	79.34	19.70
	Sig	**< 0.001**	0.061	**< 0.001**	**< 0.001**	**< 0.001**	0.360	**< 0.001**
SVM	All	66.90	68.38	70.76	88.82	95.01	68.59	21.35
	Selected	90.44	73.76	88.02	87.58	94.67	77.28	19.96
	Sig	**< 0.001**	0.118	**< 0.001**	**< 0.001**	**< 0.001**	**0.003**	0.877
j48	All	94.97	86.20	89.53	83.02	92.65	81.83	20.98
	Selected	96.04	86.20	88.80	86.73	94.62	80.20	17.63
	Sig	0.021	0.417	0.082	**0.003**	0.268	0.200	0.090

Bold cells show significant differences after correction for multiple tests.

Table 12 shows the common features selected across populations by Relief-F. Despite comparing populations with a larger subset of ten features, less than three features were common between populations.

**Table 12:** Common features ranked in the top ten across populations using Relief-F.

Level 1	Level 2	Level 3	Level 4	Level 5	Level 6	Level 7
**Able bodied and Senior**						
15	66,	76, 62	66, 52	64,42	n/a	50
**Able bodied and stroke patients**						
54	12	17	35, 48, 75	59	44, 48, 65	11, 49, 55, 75
**Stroke patients and senior**						
56, 66	1,2,11	57, 64	14, 63	n/a	57	15, 44, 72
**All three populations**						
n/a	n/a	n/a	n/a	n/a	n/a	n/a

### Analysis of results

#### Level 1: Mobile, immobile states

When distinguishing between mobile and immobile states, the “simple moving average of sum of range of linear acceleration” (feature 3), “simple moving average of the sum of variances of the gravity vector” (feature 23), mean gyroscope output on the *y* and *z* axes (features 70, 71), and correlation between acceleration in gravity and heading direction (feature 66) were selected by multiple algorithms across populations.

The CFS method selected features 3 and 23 for all populations and FCBF selected feature 3 for both able bodied and stroke populations. Both of these features are moving averages, which filter the raw signal to provide a more consistent, smoothed feature. Filtering makes the features more effective in differentiating between states at a broad level of detail; however, these features were not selected for the higher detail levels.

Mean gyroscope output on the *y* and *z* axes (features 70, 71) were selected by CFS for all populations. Since many HAR systems employ only accelerometers, informative gyroscope data are unavailable for activity classification. Most commercial smartphones have gyroscopes built in, making gyroscope data accessible and feasible.

Feature 66 was selected by two algorithms for each population, but without consistent results, and examines the relationship between vertical and horizontal accelerations. This feature has been used to differentiate between activities that translate along a single axis, such as walking, from activities that translate along multiple axes, such as stair climbing [18]. Since acceleration in an immobile state would exist predominantly in one direction, between axis correlations would be small, which explains why feature 66 was selected at level 1. Interestingly, feature 66 was not selected for all groups. Some people may perform mobile activities, such as walking with pathological gait, with smaller correlations between acceleration axes (i.e., mobile and immobile states both having smaller correlations), which would result in this feature not being consistently selected across populations.

#### Level 2: Sit, stand and Level 3: Sit, stand, lie

When distinguishing between sitting, standing, and lying down, feature 10 (mean *z* gravity vector) and feature 4 (difference to *y* gravity) were repeatedly selected across populations.

For differentiating sitting and standing, the CFS method selected feature 10 for all populations. This feature is related to the phone’s orientation, thus feature 10 is a reasonable choice for differentiating between sitting and standing since the pelvis angle changes during these activities. This feature was selected by FCBF for able bodied and senior participants, but not stroke. The mean *y*-gravity vector also changes with phone orientation; however, these changes are small in comparison to the initial value in an upright position (roughly 9.81 m/s^2^). This small change would not have been identified as significant, which could be why the mean *y* gravity vector was not selected repeatedly, as opposed to mean *z* gravity that was near zero when upright.

Interestingly, feature 4 (difference to *y* gravity) was selected for all populations by CFS when including “Lie” as a class (level 3), even though feature 4 was only selected for the senior population when differentiating solely between sit and stand (level 2). This suggests that, if the pelvis remains relatively upright for sit and stand, the *z*-axis gravity vector (feature 10) is better at differentiating between these smaller changes in phone orientation than feature 4. For level 3, FCBF selected feature 4 for able bodied and senior populations, but not stroke patients.

For the Relief-F subset, no features were selected for all populations when comparing sitting, standing and lying (level 3). The features that ranked well were those that examined a single acceleration axis (cumulative sum of *y* linear acceleration, skewness of *z* acceleration, kurtosis of *x*-gravity etc.). Since these activities affect pelvis orientation, a combination of features examining behaviour along different axes can indicate the person’s state. Interestingly, the features selected by Relief-F were not similar to the ones selected by CFS and FCBF at level 3. When implementing these features in a HAR system, the CFS and FCBF subsets could be considered first since these selection algorithms take feature redundancy into consideration.

#### Level 4: Large movements, stairs

When distinguishing stair climbing from other large movements, four features were selected for all populations by the CFS method: feature 4 (difference to *y* gravity), feature 24 (maximum slope of simple moving average of sum of variances), feature 29 (element (1, 3) of the gravity vector’s covariance matrix) and Feature 52 (moving average of the skewness of the rotated *y* linear acceleration). Feature 52 was also selected for all populations by FCBF.

Feature 4 relates to pelvis orientation. A person who walks upright, but leans forward when navigating stairs could exhibit a change in pelvis orientation. Feature 24 (maximum slope of simple moving average of sum of variances) describes how the acceleration variance increases or decreases, which would change if the person slows down or speeds up while climbing stairs. Feature 29 describes how the variance along different axes change together, and feature 52 describes the asymmetry of a person’s vertical acceleration, making them viable features to identify the difference in the direction of motion between stair climbing and walking.

#### Level 5: Ramp up, ramp down, large movements, stairs up, stairs down

Similarly to Level 4, feature 4 (difference to *y* gravity) and feature 29 (element (1, 3) of the gravity vector’s covariance matrix) were selected by CFS for all populations. These features describe how the acceleration axes relate to each another, which is directly affected by changes in pelvis orientation and the direction of movement (i.e., if a person moved up a staircase or ramp). FCBF did not select any features that were common to all populations, although features 4 and 29 were selected for two populations each, agreeing with CFS selections. Interestingly, level 5 accuracy tended to be better than level 4. Therefore, different features should be used to classify stair ascent and descent, rather than combining ascent and descent into one class.

#### Level 6: Small movements

For all populations, feature 10 (mean *z* gravity vector) and feature 70 (mean gyroscope on *y*-axis) were selected by CFS. Since these classes describe when a person is slightly moving while seated, standing, or lying down, these motions can be characterized by pelvis rotation, as measured by the gyroscope. The mean *z* gravity vector is along the phone’s forward axis, which changes when a person leans in to accomplish a small movement task, such as making toast or eating dinner.

#### Level 7: Transitions

No features were common to all populations when classifying transitions. Due to the short duration of transition states and movement variability between individuals, it is difficult to identify consistent features across multiple people or groups. Since transitions are defined by the two activities performed at the start and end of the transition period, other methods could be considered to classify a transition (i.e., without using specific transition features).

## Discussion

Smartphone signal features that were consistent across able-bodied, elderly, and stroke groups were successfully identified. This established viable HAR feature subsets that can be used with waist-worn smartphones. Evaluation of these subsets with generic classifiers showed improvements in activity recognition accuracy. This indicates that the features eliminated in the feature selection process were redundant and did not significantly contribute to classifier accuracy. Thus, with appropriate feature subset selection, equivalent classifier performance can be obtained with a reduced feature set, effectively reducing computation burden on the HAR.

When differentiating between sitting, standing, and lying down, the mean gravity signal along the phone’s *z*-axis was repeatedly selected across populations. This is similar to results obtained by Cruz-Silva et al. [20], who ranked mean horizontal acceleration third and mean *z* acceleration 15^th^ out of 159 features for HAR (gravity vector was not included). For our study, the feature selection algorithms found the gravity signal to be more relevant, and the redundant mean acceleration signal was therefore excluded (i.e., the gravity signal along the *z*-axis and mean horizontal acceleration may be similar for the target activities). Maurer et al. [11] also selected mean acceleration in the *z*-axis for HAR, although neither Cruz-Silva nor Maurer separated their data into different levels. The mean *z*-axis gravity feature is related to the phone’s orientation, which changes as the pelvis rotates when transitioning between immobile states, such as sitting or lying down. The mean *z* gravity feature was not selected by FCBF for the stroke group, who may have a different posture when standing that lead to incorrect classification as sitting [29].

Skewness (asymmetry) of the rotated linear acceleration along the phone’s *y*-axis was selected by CFS and FCBF for differentiating between large movements and stairs. This result is supported by Hache et al. [30] who identified vertical skewness as a viable feature for identifying stair ascent or descent. In future work, other activities that produce vertical acceleration (i.e., jumping, hopping, jogging, etc.) could be included to verify if skewness would remain a viable feature. Acceleration covariance was frequently selected when detecting ramp and stair ascent or descent. This feature measures how acceleration axes change together, which is affected by the person’s orientation as they move on an incline.

Differentiating between stair navigation and walking is difficult for HAR systems that only use one sensor location. Pelvis movements are similar for both activities, making it difficult to derive useful information from motion sensors such as accelerometers and gyroscopes. Classification accuracy is typically lower for stair recognition than other activities when using HAR systems with a single sensor location, such as a smartphone [9,31,32]. This is supported by the lower classification accuracies found at level 4.

While this research identified features that were commonly selected across populations, diversity between populations did occur for many features. For example, features selected for the stroke group tended to differ from the able-bodied and elderly groups. This may be due to the inhomogeneity of the stroke participants, whose mobility levels varied and some people used crutches and arm slings. Nine stroke patients had right hemiparesis, thereby reducing pelvis movement on the right side where the phone was attached, affecting sensor and feature output. Also, many stroke participants attached the holster to cotton pants that had an elastic waist strap, which may have provided an inferior anchor point compared to the leather belts and fitted pants that the able bodied and elderly populations typically wore. This may increase sensor signal variability for stroke participants and demonstrates the importance of including the target group when training and evaluating HAR systems, so that a classifier is not tailored to an inappropriate sample set.

The highest accuracies were achieved using feature subsets selected by the CFS algorithm. Some limitations exist in the current study. While the feature selection methods were designed to compensate for class imbalances in the feature set, selective sampling before performing feature selection could improve results in future work. The classifiers were used to evaluate the quality of the selected feature subsets; however, they were not customized to the specific HAR application. When implementing the features selected in this study in HAR classifiers, it is recommended that the classifier be tailored to the specific needs of the situation. Three separate populations were included in this study, but the total sample set was small at 44 participants. Larger data sets could contribute to future work.

## Conclusion

This research selected smartphone signal feature subsets for human activity recognition that were applicable across able bodied, elderly, and stroke populations. Three filter-based feature selection methods were used so that identification of useful features could be performed independent of the classifier. This information can guide future smartphone-based HAR system development among the targeted users, regardless of the classifier. In particular, the following signal features were effective across multiples populations: (i) acceleration features using simple moving averages and correlations of acceleration along different axes, as well as mean gyroscope output on the *y* and *z* axes, to distinguish between immobile and mobile states; (ii) gravity signal mean in the phone’s forward direction and the difference between the phone’s *y* gravity to the *x* and *z* gravity signals when distinguishing between sit, stand, and lie; (iii) the gravity signal range in the forward direction for differentiating between sitting and standing; (iv) skewness of the rotated linear acceleration along the *y*-axis for classifying stair climbing from other large movements; (v) acceleration covariance when detecting ramp and stair ascent or descent; (vi) mean gyroscope signal on the *y*-axis for detecting small movements.

Future research could expand on the study results through feature selection with different pathological populations, such as amputees or people with neurological disorders, since different gait patterns may identify additional features to be included in a generalized feature set.

## Supporting Information

S1 Appendix Transition classes. (DOCX)
