# Supplementary material for: Feature Selection for Wearable Smartphone-Based Human Activity Recognition with Able bodied, Elderly, and Stroke Patients
Source: PLoS One. 2015 Apr 17;10(4):e0124414. doi: 10.1371/journal.pone.0124414 (PMC4401457; doi:10.1371/journal.pone.0124414)
Supplement: S1 Appendix — (DOCX) [file pone.0124414.s001.docx]

# S1 Appendix

Transition classes

| 1 | stand-sit (1-2) |
| --- | --- |
| 2 | stand-lie (1-3) |
| 3 | Stand-walk (1-4) |
| 4 | Stand-small move (1-6) |
| 5 | Sit-stand (2-1) |
| 6 | Sit-lie (2-3) |
| 7 | Sit-walk (2-4) |
| 8 | Lie-stand (3-1) |
| 9 | Lie-sit (3-2) |
| 10 | Lie-walk (3-4) |
| 11 | Walk-stand (4-1) |
| 12 | Walk-sit (4-2) |
| 13 | Walk-lie (4-3) |
| 14 | Walk-stairs (4-5) |
| 15 | Walk-small move (4-6) |
| 16 | Stairs-walk (5-1) |
| 17 | Stairs-walk (5-4) |
| 18 | Small move-stand (6-1) |
| 19 | Small move-sit (6-2) |
| 20 | Small move-lie (6-3) |
| 21 | Small move-walk (6-4) |
